# Effect of Compression Loading on Human Nucleus Pulposus-Derived Mesenchymal Stem Cells

**DOI:** 10.1155/2018/1481243

**Published:** 2018-10-08

**Authors:** Hang Liang, Sheng Chen, Donghua Huang, Xiangyu Deng, Kaige Ma, Zengwu Shao

**Affiliations:** Department of Orthopaedics, Union Hospital, Tongji Medical College, Huazhong University of Science and Technology, 1277 Jiefang Avenue, Wuhan 430022, China

## Abstract

**Purpose:**

Mechanical loading plays a vital role in the progression of intervertebral disc (IVD) degeneration, but little is known about the effect of compression loading on human nucleus pulposus-derived mesenchymal stem cells (NP-MSCs). Thus, this study is aimed at investigating the effect of compression on the biological behavior of NP-MSCs in vitro.

**Methods:**

Human NP-MSCs were isolated from patients undergoing lumbar discectomy for IVD degeneration and were identified by immunophenotypes and multilineage differentiation. Then, cells were cultured in the compression apparatus at 1.0 MPa for different times (0 h, 24 h, 36 h, and 48 h). The viability-, differentiation-, and differentiation-related genes (Runx2, APP, and Col2) and colony formation-, migration-, and stem cell-related proteins (Sox2 and Oct4) were evaluated.

**Results:**

The results showed that the isolated cells fulfilled the criteria of MSC stated by the International Society for Cellular Therapy (ISCT). And our results also indicated that compression loading significantly inhibited cell viability, differentiation, colony formation, and migration. Furthermore, gene expression suggested that compression loading could downregulate the expression of stem cell-related proteins and lead to NP-MSC stemness losses.

**Conclusions:**

Our results suggested that the biological behavior of NP-MSCs could be inhibited by compression loading and therefore enhanced our understanding on the compression-induced endogenous repair failure of NP-MSCs during IVDD.

## 1. Introduction

Intervertebral disc (IVD) degeneration is among the most important contributors to low back pain, leading to patient disability and heavy financial burdens globally [1, 2]. Currently, conservative and surgical operations are the main treatments for IVD degeneration. However, these treatments are not long-lasting and effective for the limitation that they cannot reverse the structural and mechanical function of IVD tissues [3]. Stem cell-based therapies have shown an exciting perspective for IVD repair recently [4]. In different animal models of disc degeneration, which are established by annular puncture or nucleus aspiration, transplantation of exogenous mesenchymal stem cells (MSCs) has improved the evaluation scores of radiographs, magnetic resonance images (MRI), and histological analysis [5–7]. In a pilot study [8], ten patients suffering from chronic back pain and positively diagnosed with lumbar disc degeneration were treated by injecting autologous expanded bone marrow MSCs into the nucleus pulposus (NP) area. The results indicated the feasibility, safety, and clinical efficacy of the treatment.

Apart from exogenous stem cell transplantation, endogenous stem cell stimulation and recruitment are also essential ways to repair IVD degeneration and play a key role in endogenous repair [9]. Evidence has been found in latest researches that nucleus pulposus mesenchymal stem cells (NP-MSCs) exist naturally in the IVD [10, 11] and participate in IVD regeneration [9]. The aim of NP-MSC therapy is to make NP-MSCs differentiate into nucleus pulposus-like cells and stimulate disc cells maintaining IVD homeostasis. Although activating the endogenous NP-MSCs could be an attractive strategy for endogenous repair, it is hard to maintain the number of viable and functional NP-MSCs under an adverse microenvironment in IVD [12]. It was reported that the viability and proliferation rate of NP-MSCs were significantly inhibited under hypoxia [13], and acidic conditions could decrease the extracellular matrix (ECM) synthesis and stem cell-related gene expression of NP-MSCs [14]. Mechanical loadings [15], including compression, shear, torsion, and flexion, are another essential factors that influence the fate of NP-MSCs.

The IVD functions as a shock absorber, and external forces on the spine lead to intense stresses that act on the IVD. From a mechanical point of view, disc cells and progenitor cells embedded in the different areas are exposed to wide ranges of mechanical loadings [16]. Inappropriate or excessive compressive force stimulus applied to intervertebral discs (IVDs) is an important contributing factor in the progress of disc degeneration. We have reported that apoptosis and necroptosis could be induced by compression at a magnitude of 1 MPa in rat NP cells previously [17, 18]. However, to our best knowledge, there have been no studies focusing on the effect of compression loading on human NP-MSCs so far. Therefore, the present study is aimed at exploring the effect of compression on the biological behavior of NP-MSCs in vitro.

## 2. Methods

### 2.1. Isolation and Culture of NP-MSCs

NP tissues were donated by five patients undergoing lumbar discectomy for lumbar disc hernia, and the ages of those five patients are 42, 49, 45, 41, and 40, respectively. According to Pfirrmann's MRI (T2WI) Grading Criteria for Disc Degeneration, all the patients were in grade III. All procedures in the present study were approved by the ethics committee of Tongji Medical College of Huazhong University of Science and Technology. NP-MSCs were isolated and cultured as previously described [14]. Briefly, NP tissues were first carefully separated by a dissecting microscope and washed by PBS solution. Secondly, the NP samples were dissected and digested in 0.2% type II collagenase for 12 h at 37°C with 5% CO_2_. And then the obtained cells and partially digested tissues were cultured in MSC complete medium (Cyagen, USA) at 37°C in a humidified atmosphere containing 5% CO_2_. The media were changed twice a week, and the primary culture was 1 : 3 subcultured when cells reached 80%–90% confluence. NP-MSCs in passage 2 were used in this study.

### 2.2. Surface Marker Identification of NP-MSCs

The collected cells were washed and resuspended in PBS and incubated with the following monoclonal antibodies according to the recommendations of ISCT [19]: CD105, CD73, CD90, CD34, CD14, CD19, and HLA-DR. After being incubated for 30 min at 37°C, the cells were washed with PBS and then resuspended in 500 *μ*L PBS to adjust the cell concentration at about 10^6^/mL. The labeled cells were examined via flow cytometry (BD LSR II, Becton Dickinson) following standard procedures.

### 2.3. Multilineage Differentiation

To assess the multilineage differentiation potential of NP-MSCs, the osteogenic, adipogenic, and chondrogenic differentiation was induced.

For osteogenic differentiation, NP-MSCs were seeded in six-well plates at 2 × 10^4^ cells/cm^2^ in normal medium and incubated in osteogenic differentiation medium (Cyagen, USA) at 60%–70% confluence. The inducing conditional medium was changed twice a week. After differentiating for 14 days, the NP-MSCs were used to extract RNA and stained with the Alizarin Red solution, respectively.

For adipogenic differentiation, NP-MSCs were seeded in six-well plates at 2 × 10^4^ cells/cm^2^. When the cells grew up to 100% confluence, the medium was changed to adipogenic differentiation medium A (Cyagen, USA). Three days later, the medium was changed to adipogenic differentiation medium B (Cyagen, USA). After 24 h, the medium was replaced back with medium A. The cycle was repeated for 4 times, and the cells were cultured in medium B for additional 7 days. After differentiating, the NP-MSCs were used to extract RNA and stained with Oil Red O.

For chondrogenic differentiation, NP-MSCs were seeded in six-well plates at 2 × 10^4^ cells/cm^2^ in normal medium and incubated in chondrogenic differentiation medium (Cyagen, USA) at 60%–70% confluence. The media were changed every 2 to 3 days. After differentiating for three weeks, the NP-MSCs were used to extract RNA and stained with the Alcian blue.

In addition, to evaluate the effect of compression loading on the multilineage differentiation of NP-MSCs, the cells were cultured in a custom-made compression apparatus for different times (0 h, 24 h, 36 h, and 48 h) at a magnitude of 1 MPa as described previously [17, 20]. And the cells were then digested with 0.25% trypsin and seeded in six-well plates for the induced differentiation above.

### 2.4. Cell Viability Assay (CCK-8)

NP-MSCs were seeded in 96-well plates and cultured in the compression apparatus for different times (0 h, 24 h, 36 h, and 48 h). Cell viability was measured by CCK-8 (Dojindo, Japan) following the manufacturer's protocol. At the appropriate time points, 10 *μ*L CCK-8 solution was added to each well. The plates were then incubated at 37°C with 5% CO_2_ for 2 h. The surviving cell counts were determined by absorbance detection at 450 nm with a spectrophotometer (BioTek, USA).

### 2.5. Colony Formation Assay

To demonstrate the effect of compression loading on the capacity of colony formation, the NP-MSCs were treated with compression for different times (0 h, 24 h, 36 h, and 48 h). Then, the cells were collected and seeded in six-well plates at the density of 200 cells/well. The medium was changed twice a week. After two weeks, the cells were fixed with 4% paraformaldehyde. Fifteen minutes later, the cells were washed with PBS and stained with 0.1% crystal violet for 15 min. The colonies containing more than 100 cells were counted.

### 2.6. Wound Healing Assay

For the wound healing assay, NP-MSCs were seeded in six-well plates with MSC complete medium. When the cells grew to 100% confluence, the wound was created by scraping the monolayer cell with a pipette tip, and the medium was replaced with serum-free DMEM-L. Then, the plates were randomly assigned to the compression group or the control group. The photomicrographs were acquired at different time points (0 h, 24 h, 36 h, and 48 h). The migration areas of wound healing were quantified using ImageJ software.

### 2.7. Transwell Migration Assay

The 24-well plates with 8 *μ*m pore-size transwell inserts were used to assess the migration abilities of NP-MSCs. The cells were adjusted to a density of 10^5^ cells/mL with serum-free DMEM-L, and then 200 *μ*L cell suspension was added into the upper chamber and 600 *μ*L DMEM-L with 10% FBS was added into the lower chamber. Subsequently, the cells were treated with compression for different times (24 h, 36 h, and 48 h), and the cells without compression treatment were used as the control. At the appropriate time points, the nonmigrated NP-MSCs were removed and the migrated cells were fixed and stained with crystal violet. The migrated NP-MSCs were counted in five randomly selected optical fields.

### 2.8. Quantitative Real-Time Polymerase Chain Reaction (QRT-PCR)

Total RNA was extracted from NP-MSCs by TRIzol reagent (Invitrogen, USA); then, the RNA was transcribed into cDNA by a reverse transcription kit (Takara,) following the manufacturer's protocol. After reverse transcription, QRT-PCR was performed with the SYBR Premix Ex Taq II according to the manufacturer's instructions (Takara). The 2^−△△CT^ method was used to analyze the data, and the housekeeping gene GAPDH was used to normalize the level of mRNA. Primer sequences were as follows: 5′-GTGGACGAGGCAAGAGTTTCA-3′ (forward) and 5′-GGTGCAGAGTTCAGGGAGGG-3′ (reverse) for RUNX2, 5′-CCCATCCCCACTTTGTGATT-3′ (forward) and 5′-ATTCCGCAGGGCAGCAAC-3′ (reverse) for APP, 5′-AGCATTGCCTATCTGGACGAA-3′ (forward) and 5′-GTACGTGAACCTGCTATTGCC-3′ (reverse) for COL2A1, and 5′-AATCCCATCACCATCTTCCAG-3′ (forward) and 5′-GAGCCCCAGCCTTCTCCAT-3′ (reverse) for GAPDH.

### 2.9. Western Blot Analysis

NP-MSCs were lysed on ice using standard buffer (Beyotime, China), and total protein was extracted by a protein extraction kit (Beyotime, China). The cell lysate was centrifuged at 12,000 ×g for 10 min at 4°C. After protein transfer, the membranes were blocked by nonfat milk and then incubated overnight at 4°C with a rat polyclonal antibody against cleaved Sox2, Oct4, and GADPH (Abcam, 1 : 3000). After washing several times, the membrane was incubated with secondary antibodies for 1 h at room temperature. Finally, the immunoreactive membranes were visualized via the enhanced chemiluminescence (ECL) method following the manufacturer's instructions (Amersham Biosciences, USA).

### 2.10. Statistical Analysis

All measurements were performed at least three times. The data were presented as mean ± standard deviation (SD). Student's *t*-tests were used in the analysis of two-group parameters. One-way analysis of variance (ANOVA) test was used in comparisons of multiple sets of data. *p* < 0.05 were considered statistically significant.

## 3. Results

### 3.1. Identification of NP-MSCs

**Figure 1:**
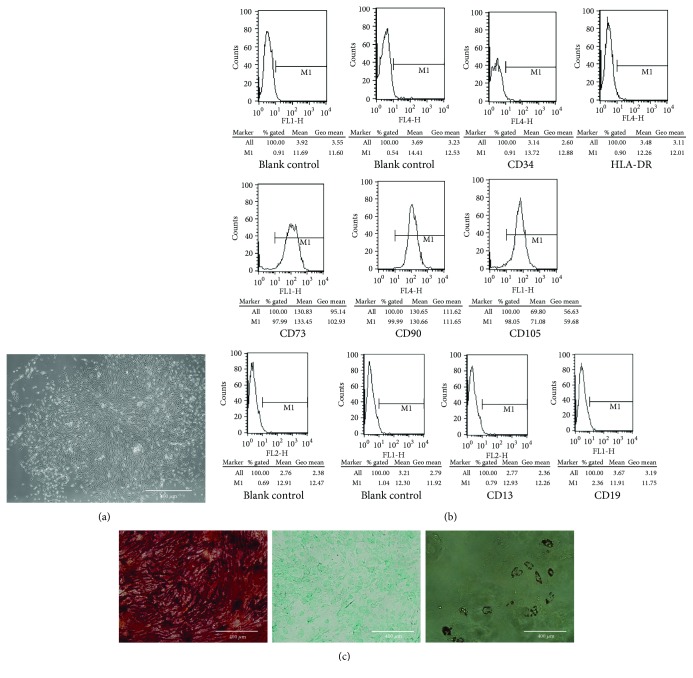
The isolated cells fulfilled the criteria of MSC stated by ISCT. (a) The shape of cells isolated from the degenerated IVDs. (b) The MSC-associated surface markers (CD34, CD14, CD19, HLA-DR, CD73, CD90, and CD105) were analyzed by flow cytometry. (c) The induction of osteogenic, chondrogenic, and adipogenic differentiation of the isolated cells.

The cells isolated from the degenerated IVDs presented with long spindle-shaped adherent growth and grew in a spiral formation (Figure 1(a)). The MSC-associated surface markers were analyzed by flow cytometry. As shown in Figure 1(b), the isolated cells had high expression levels of markers (CD105, CD73, and CD90) that are normally positive in MSCs and had low expression of markers (CD34, CD14, CD19, and HLA-DR) that are usually negative in MSCs. For osteogenic differentiation, the cells formed highly visible calcium deposits after being induced in osteogenic media for two weeks. In addition, oil droplets were accumulated in the cells and stained with Oil Red O during the adipogenic differentiation. After being induced in chondrogenic media, the cells exhibited strong production of sulfated proteoglycans (Figure 1(c)). The results above showed that the isolated cells fulfilled the criteria of MSC stated by ISCT. Thus, we isolated NP-MSCs, and the cells in passage 2 were used in this study.

### 3.2. Compression Loading Inhibited the Viability

**Figure 2:**
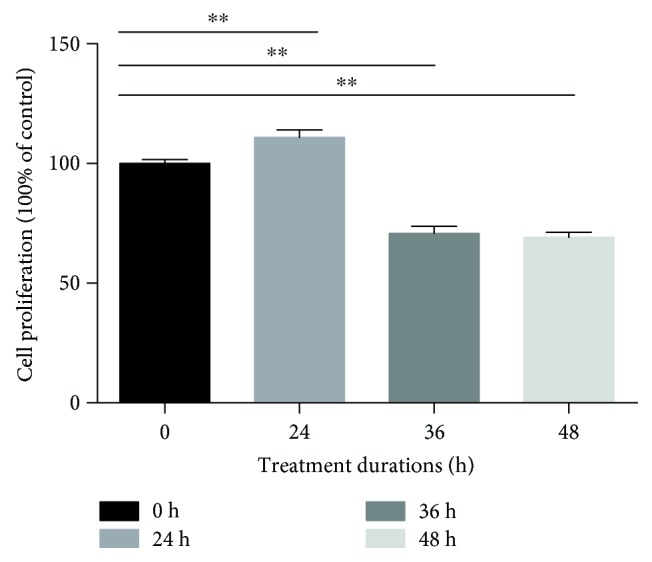
CCK-8 assay showed compression loading inhibiting the viability of NP-MSCs (values are presented as means ± SD, ^∗∗^*p* < 0.01 versus control).

To evaluate the effect of the compression loading on the viability of NP-MSCs, a CCK-8 assay was performed. As observed in Figure 2, the viability of the NP-MSCs was inhibited by compression and the inhibiting effect was significantly increased as compression time was prolonged except for the time of 24 h, which has shown an increased cell viability. A possible explanation of this phenomenon is that moderate compression may increase the cell viability, but this improvement cannot contribute to the stemness ability of intervertebral disc stem cells (Figure 2, values are presented as means ± SD, ^∗^*p* < 0.05 and ^∗∗^*p* < 0.01 versus control).

### 3.3. Compression Inhibited the Multilineage Differentiation Potential of NP-MSCs

**Figure 3:**
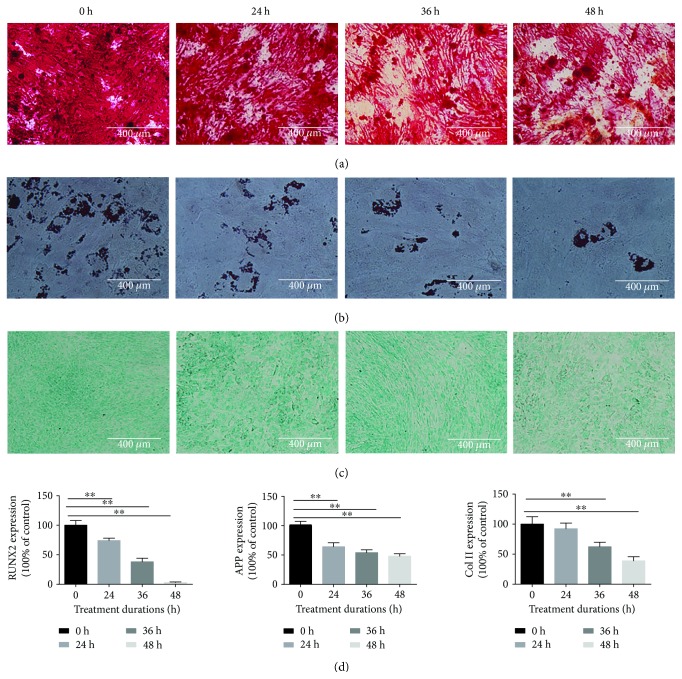
Compression inhibited the multilineage differentiation potential of NP-MSCs. (a) The induction of osteogenic differentiation under the increased compression time from 0 h to 48 h. (b) The induction of adipogenic differentiation under the increased compression time from 0 h to 48 h. (c) The induction of chondrogenic differentiation under the increased compression time from 0 h to 48 h. (d) The expressions of osteogenesis genes (Runx2), adipocyte-specific genes (APP), and chondrocyte-specific genes (Col2) under the increased compression time from 0 h to 48 h (values are presented as means ± SD, ^∗∗^*p* < 0.01 versus control).

To determine the effect of the compression loading on the multilineage differentiation potential of NP-MSCs, the osteogenic, adipogenic, and chondrogenic differentiation was induced and the mRNA expressions of Runx2, APP, and Col2 for the osteogenic, adipogenic, and chondrogenic differentiation, respectively, were analyzed. The results showed that the multilineage differentiation was suppressed in accordance with the increased compression time from 0 h to 48 h. For osteogenic differentiation, the mineralized nodules and the expressions of osteogenesis genes (Runx2) significantly decreased in the NP-MSCs with compression treatment (Figures 3(a) and 3(d)). For adipogenic differentiation, the accumulated lipid vacuoles decreased and the expression of adipocyte-specific genes (APP) significantly downregulated (Figures 3(b) and 3(d)). For chondrogenic differentiation, the proteoglycans and the expressions of chondrocyte-specific genes (Col2) obviously decreased (Figures 3(c) and 3(d)). (Figure 3, values are presented as means ± SD, ^∗^*p* < 0.05 and ^∗∗^*p* < 0.01 versus control).

### 3.4. Compression Inhibited the Capacity of Colony Formation

**Figure 4:**
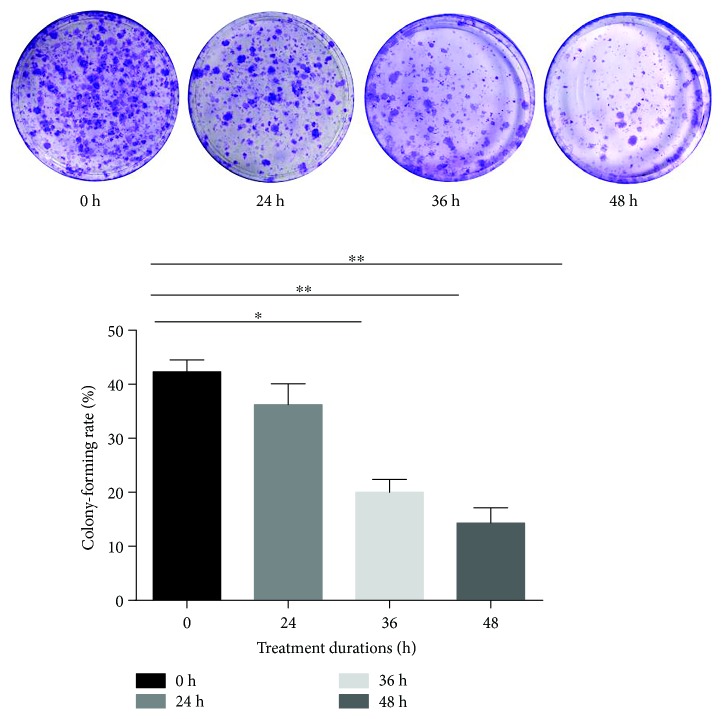
Compression inhibited the capacity of colony formation (values are presented as means ± SD, ^∗^*p* < 0.05 and ^∗∗^*p* < 0.01 versus control).

The colony-forming ability is a vital factor to evaluate the stemness of NP-MSCs. In the control group, the colony-forming rate was 46%. But in the compression group, the results demonstrated that the colony-forming rate was inhibited by 43.33%, 38.27%, 19.4%, and 14.33% compared to the control at different time points from 24 h to 48 h (Figure 4, values are presented as means ± SD, ^∗^*p* < 0.05 and ^∗∗^*p* < 0.01 versus control).

### 3.5. Compression Inhibited the Migration Ability

**Figure 5:**
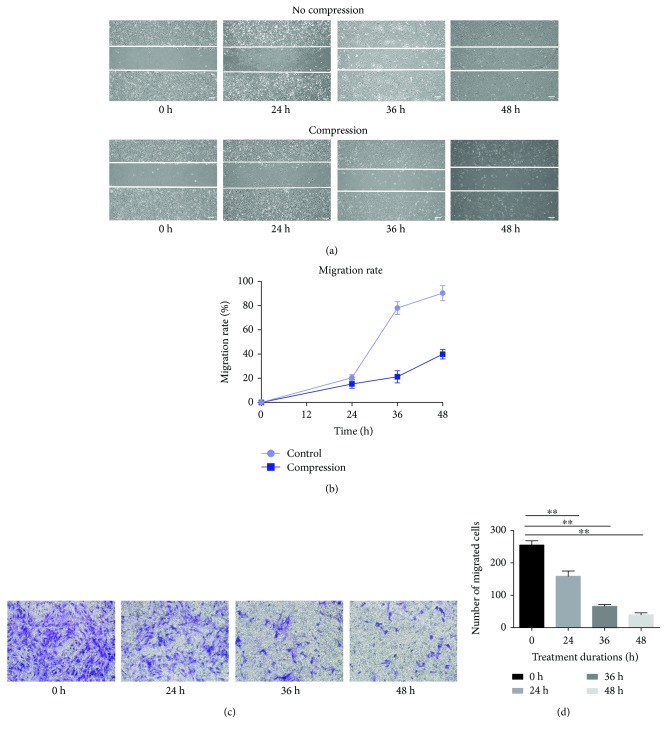
Compression inhibited the migration ability. (a, c) The assessment of nondirectional migration ability by wound healing assay with or without compression from 0 h to 48 h. (b, d) The assessment of directional migration ability by transwell migration assay under compression from 0 h to 48 h (values are presented as mean ± SD, ^∗∗^*p* < 0.01 versus control).

Nondirectional migration ability and directional migration ability were assessed by wound healing assay and transwell migration assay, respectively. As shown in Figure 5, the migration areas and the migrated NP-MSCs were increased over time. However, the migration areas and the migrated NP-MSCs significantly decreased compared to those at different time points from 24 h to 48 h (Figure 5, values are presented as means ± SD, ^∗^*p* < 0.05 and ^∗∗^*p* < 0.01 versus control).

### 3.6. Compression Decreased the Expression of Stem Cell-Related Genes (Sox2 and Oct4)

**Figure 6:**
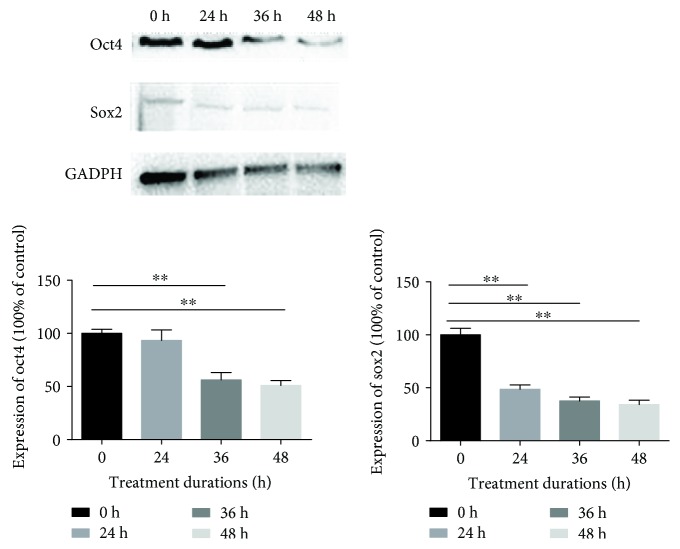
Compression decreased the expression of stem cell-related genes (Sox2 and Oct4). The expression of Sox2 and Oct4 was analyzed by Western blotting and normalized against GAPDH levels (values are presented as mean ± SD, ^∗∗^*p* < 0.01 versus control).

The genes, including Sox2 and Oct4, were considered the specific genes that maintained the stemness of MSCs. The expression of proteins (Sox2 and Oct4) was significantly decreased. Protein levels of Sox2 and Oct4 from treated cell lysates were analyzed by Western blotting and normalized against GAPDH levels (Figure 6, values are presented as means ± SD, ^∗^*p* < 0.05 and ^∗∗^*p* < 0.01 versus control).

## 4. Discussion

Endogenous stem cell repair for IVD degeneration provides a novel strategy to reverse the structure and mechanical function of IVD tissues, and it has excited a great interest to scientists in the past decade. In 2007, Risbud et al. [21] isolated and identified the skeletal progenitor cells from degenerate human disc in vitro, which could express a series of specific surface markers of MSCs and commit to multilineage differentiation. In 2009, Henriksson et al. [22] applied the labeling technique in vivo and demonstrated the existence of slow-cycling cells and stem cell niche in the IVD region. After that, many groups isolated and identified MSCs from the nucleus pulposus, annulus fibrosus, and cartilage endplate [10, 11, 23–30]. In the present study, the cells isolated from NP had the following characteristics: (1) the cells presented with long spindle-shaped adherent growth and grew in a spiral formation; (2) the cells were positive for CD105, CD73, and CD90 and negative for CD34, CD14, CD19, and HLA-DR; and (3) the cells had the multilineage differentiation potential. These characteristics met the criteria stated by the ISCT for MSC.

In the progress of endogenous tissue regeneration, a single stem cell produces two daughter cells: one maintains the stemness to renew itself while the other differentiates to a specific cell to conduct endogenous repair [31]. In terms of endogenous NP-MSC repair for IVD degeneration, one part of NP-MSCs retains the stem cell identity, and another part of NP-MSCs differentiates into nucleus pulposus-like cells and stimulus disc cells to repair the degenerative disc. Therefore, sustaining the number of viable and functional NP-MSCs is vital for maintaining the IVD homeostasis [9]. However, the IVD is avascular and NP-MSCs in the discs have to bear various stresses, including acidity, hypoxia, nutrient deficiency, and hypertonicity and mechanical loads [12]. Although mechanical stress plays an essential role in the progression of IVD degeneration, there have been no studies reporting the effect of compression loading on human NP-MSCs so far. Thus, the current study applied a custom-made compression apparatus for the first time to study the effect of compression loading on the biological behavior of NP-MSCs in vitro.

The results showed that the viability of the NP-MSCs was inhibited by compression except for the time of 24 h, which has shown an increased cell viability. A possible explanation of this phenomenon is that moderate compression may increase the cell viability, but this improvement cannot contribute to the stemness ability of intervertebral disc stem cells. Stem cells may be a special type of intervertebral disc cells which can react to a short time of compression; however, the differentiation ability, migration ability, and the expression of stem protein have shown a damage when the cells were exposed to the compression of 24 h. We have to say it is interesting and a little strange, but we all know that cell viability cannot be the representative sign of other abilities so this phenomenon needs further research. What is more, the multilineage differentiation potential was also inhibited by compression. But Dai et al. and Kim et al. reported that dynamic compression promoted the proliferation and differentiation of exogenous mesenchymal stem cells [32, 33]. We considered that the conflicting results mainly came from the different modes of compression exerted on cells and the distinct cell types used in the study. In the present study, 1.0 MPa and continuous compression was applied, and NP-MSCs isolated from degenerative NP tissue were used. However, in the study of Dai et al. and Kim et al., lower (17 kPa, 0.2 MPa) and intermittent compression was applied, and viable exogenous MSCs isolated from adipose tissue or bone marrow were used. It was reported that the intradiscal pressure value in a healthy human is around 0.1 MPa in a prone position, 0.5 MPa in a standing position, 1.1 MPa in a flexed position, and even 2.3 MPa in a standing position carrying a weight [15, 34]. Moreover, multiple stress peaks appear under aging and degeneration environment in the IVD [15]. Therefore, our study simulated the effect of excessive compression in degenerative discs on NP-MSCs and emphasized the research of the endogenous repair failure during IVDD.

MSCs can generate colonies when plated at low densities and can also migrate to sites of injury. It has been shown that the colony-forming ability and migration ability are the important biological characteristics of MSCs [35, 36]. The results revealed that compression loading inhibited the colony-forming ability and migration ability. Stem cell-related genes and proteins (Sox2 and Oct4) also play a vital role in maintaining MSC properties [37, 38]. In keeping with our results above, compression loading suppressed the expression of Sox2 and Oct4. All results in this study suggested that inappropriate or excessive compression loading inhibited the biological behavior of NP-MSCs and might be one of the mechanisms of endogenous repair failure for IVD degeneration. Before the IVD tissue-derived MSCs were isolated, many attentions were concentrated on the fate of disc cells and the repair mainly centered around how to supply the disc cells with exogenous cells, growth factors, or biomaterial [16, 39, 40]. Little is known about endogenous stem cells and endogenous repair. The present study investigated the effect of compression on the biological behavior of NP-MSCs in vitro and tried to enhance our understanding on the endogenous repair failure for IVD degeneration. Moreover, it provided a novel method to repair IVD degeneration by activating the endogenous NP-MSCs.

In conclusion, findings from our study demonstrated that the biological behavior of NP-MSCs could be inhibited by compression loading, and it might be one of the mechanisms of endogenous repair failure for IVD degeneration. Further studies discussing the effect of compression on NP-MSCs in vivo and the underlying specific mechanism may be helpful to understand the endogenous repair failure of NP-MSCs during IVD degeneration.

## Data Availability

The data used to support the findings of this study are available from the corresponding author upon request.

## Abbreviations

NP-MSCs: Nucleus pulposus-derived mesenchymal stem cells
ISCT: International Society for Cellular Therapy
IVD: Intervertebral disc
MSCs: Mesenchymal stem cells
MRI: Magnetic resonance images
NP: Nucleus pulposus
ECM: Extracellular matrix
CCK-8: Cell viability assay
QRT-PCR: Quantitative real-time polymerase chain reaction
SD: Standard deviation
ANOVA: One-way analysis of variance.
